# Additional data of cryptic species of the blind mole rat (*Nannospalax*, Rodentia) (2n = 52, NF = 84) from the Eastern Anatolia Region of Türkiye

**DOI:** 10.3897/compcytogen.20.185104

**Published:** 2026-03-26

**Authors:** Gökhan Yürümez

**Affiliations:** 1 Biology Department, Science and Art Faculty, Batman University, Batman, Türkiye Biology Department, Science and Art Faculty, Batman University Batman Türkiye

**Keywords:** Chromosome arm number, karyotype, mammalian cryptic species, *Nannospalax* spp., NORs

## Abstract

In this study, five blind mole rat specimens of the *Nannospalax* sp. were collected from two different localities in Muş Province, Eastern Anatolia, Türkiye. Cytogenetic analyses of these specimens revealed that the diploid chromosome number, the fundamental chromosome number, and the number of autosomal arms were 2n = 52, NF = 84 and NFa = 80, respectively. The X chromosome was medium-sized and submetacentric, while the Y chromosome was small and acrocentric. Active nucleolus organizer regions (NORs) were observed on the telomeric regions of three bi-armed autosomal pairs in all examined specimens. This study presents new karyological data regarding the blind mole rat with 2n = 52 chromosomes distributed in the Eastern Anatolia region from Türkiye.

## Introduction

**Figure 1.**
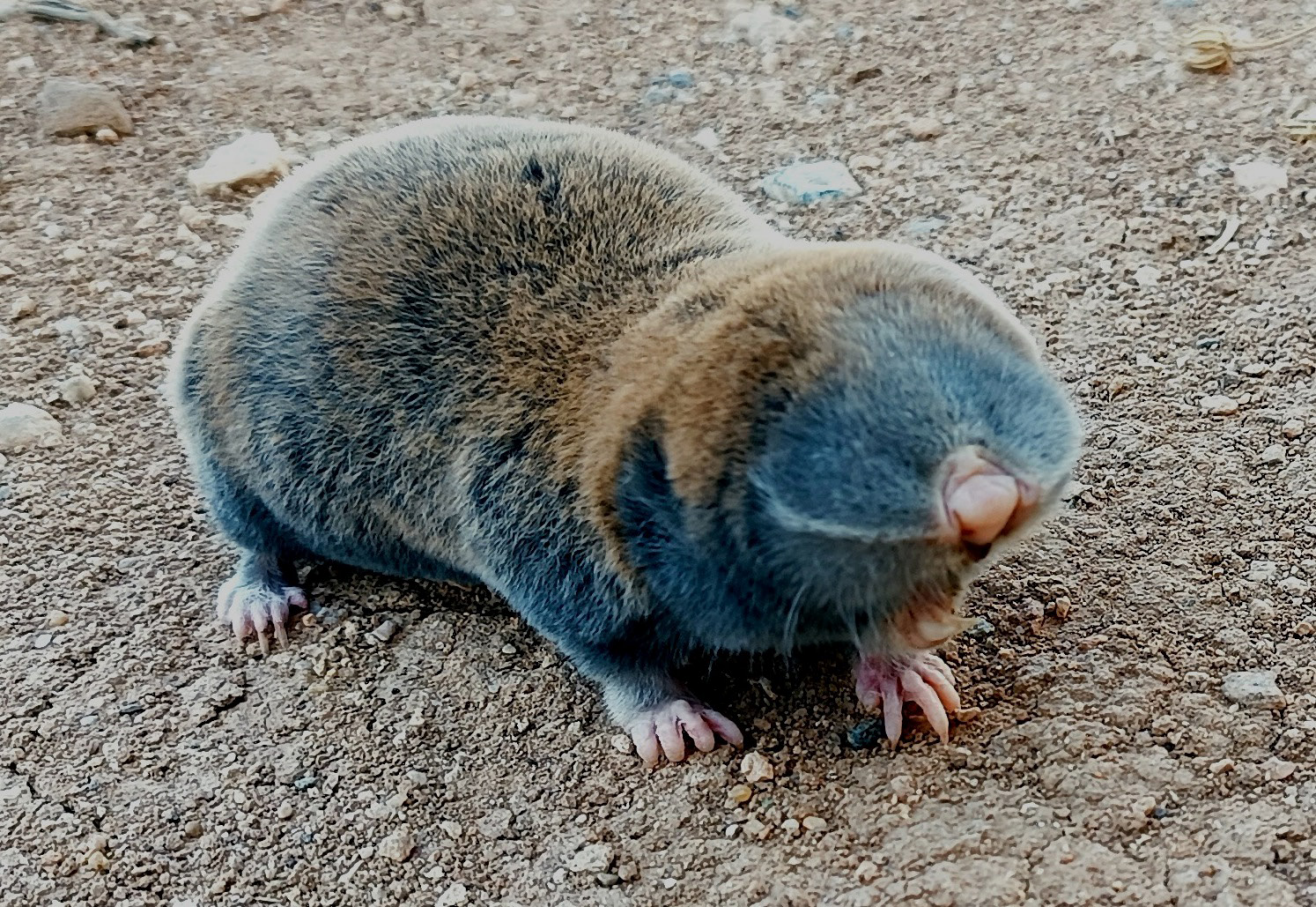
The blind mole rat specimen from Dilimli village (Muş province, Türkiye).

Species of the family Spalacidae are highly adapted to subterranean habitats, exhibiting distinctive morphological traits due to specialized burrowing ability, such as cylindrical body, short limbs, absence of external ear pinnae, vestigial tail, soft short fur, and subcutaneous eyes (Fig. 1) (Topachevskii 1969; Nevo 1979; Kryštufek and Vohralík 2009; Amr et al. 2018) as well as physiological adaptations, including strong neck muscles for digging, tolerance to hypoxia and hypercapnia (Nevo 1979; Shams et al. 2005; Nevo 2013; Coşkun et al. 2016), and heightened sensitivity to soil vibrations (Heth et al. 1987).

The taxonomic history of family Spalacidae is complex. Méhely (1909) recognized the genus *Spalax* Güldenstaedt, 1770 including three subgenera (*Microspalax* Méhely, 1909, *Mesospalax* Méhely, 1909 and *Macrospalax* Méhely, 1909), while later Ognev (1947) proposed two subgenera (*Microspalax* and *Spalax*) in the genus *Spalax*. Topachevskii (1969) classified the family Spalacidae into two genera (*Spalax* and *Microspalax*). Gromov and Baranova (1981) stated that the genus name *Microspalax* is “Nomen praeoccupatum”, “Junior homonym” and was given to a genus of the order Acarina by Megnin and Trouessart (1884). Therefore, they proposed the name *Nannospalax* Palmer, 1903 instead of *Microspalax*. Currently, the family Spalacidae is accepted as comprising two genera, *Spalax* and *Nannospalax* (Gromov and Baranova 1981; Hadid et al. 2012) and the blind mole rats in Türkiye belong to the genus *Nannospalax* (Musser and Carleton 2005; Kryštufek and Vohralík 2009). Convergent morphology and intensive chromosomal speciation within Spalacidae have divided taxonomists since this family was established (Savić et al. 2017).

Taxa of the genus *Nannospalax* are distributed in the Balkans, Southeast Europe, the Caucasus, the Middle East, Libya, Egypt and Türkiye (Topachevskii 1969; Savić and Nevo 1990). The taxonomic status of blind mole rat populations in Türkiye is also complicated. Initially, only two species (*N. leucodon* (Nordmann, 1840) and *N. ehrenbergi* (Nehring, 1898)) were recognized in the country (Mursaloğlu 1979; Kıvanç 1988; Nevo et al. 1995; Sözen et al. 2006). Some researchers suggest that *N. xanthodon* Nordmann, 1840 is found across all of Anatolia except Southeastern Anatolia and Thrace (Kryštufek and Vohralík 2009; Arslan and Zima 2014, 2015a, 2015b, 2017; Sözen et al. 2015).

The family Spalacidae is one of the most remarkable mammalian models for studying their physiology, chromosomal speciation, cryptic diversity, and karyotypic evolution. Particularly the genus *Nannospalax* has attracted considerable interest among evolutionary biologists because of its high degree of chromosomal polymorphism (Savić and Nevo 1990; Nevo 1999). The cytogenetic differences observed in blind mole rats exhibit the critical role of geographical isolation and microevolutionary processes in cryptic speciation (Savić et al. 2017).

Blind mole rats distributed throughout Türkiye, except for coastal regions, display remarkable karyotypic diversity. Conventional cytogenetic analyses of Turkish blind mole rats have identified 20 chromosomal forms: 13 in *N. xanthodon* (2n = 36, 38, 40, 44, 46, 48, 50, 52, 54, 56, 58, 60, 62), 6 in *N. ehrenbergi* (2n = 48, 52, 54, 56, 58, 60), and one in *N. leucodon* (2n = 56) (Kryštufek and Vohralík 2009; Arslan et al. 2016). When autosomal arm numbers (NF) are considered, this number exceeds 50 (Nevo et al. 1995, 2001; Ivanitskaya et al. 1997; Sözen 2004; Kankılıç et al. 2007a, 2007b, 2009, 2010, 2024a; Matur et al. 2011; Arslan et al. 2016). Populations with karyotypes 2n = 50 and 2n = 48, which are distributed in the Eastern Anatolia region of Türkiye have been identified by some researchers (Coşkun 2003; Coşkun et al. 2009, 2012; Kankılıç and Gürpınar 2014; Kankılıç et al. 2024) as *N. nehringi*.

Different karyotypic values have been reported for blind mole rats in the studied region and its surrounding areas. Specimens with a 2n = 54 (NF = 74) karyotype have been reported from Bingöl Province (Nevo et al. 1994; Coşkun 2004, and Coşkun 2010), Muş Province (Coşkun et al. 2009 and Kankılıç et al. 2024), and Bitlis Province (Coşkun et al. 2009), and these populations have been identified as *N. tuncelicus* (Coşkun 1996).

In addition, Coşkun et al. (2009) and Arslan and Zima (2017) reported the blind mole rat specimens from Malazgirt (Muş) to have a karyotype of 2n = 48 (NF = 72).

Coşkun et al. (2012) classified blind mole rat specimens from Hınıs (Erzurum) and Çat (Erzurum) as *N. nehringi* and reported that they had karyotypes of 2n = 48 (NF = 68) and 2n = 50 (NF = 70), respectively.

Coşkun (2010) reported that blind mole rat specimens collected near Bingöl–Karlıova (3 km NE) had a 2n = 52 (NF = 74) karyotype and identified these specimens as *Nannospalax* sp.

**Figure 2.**
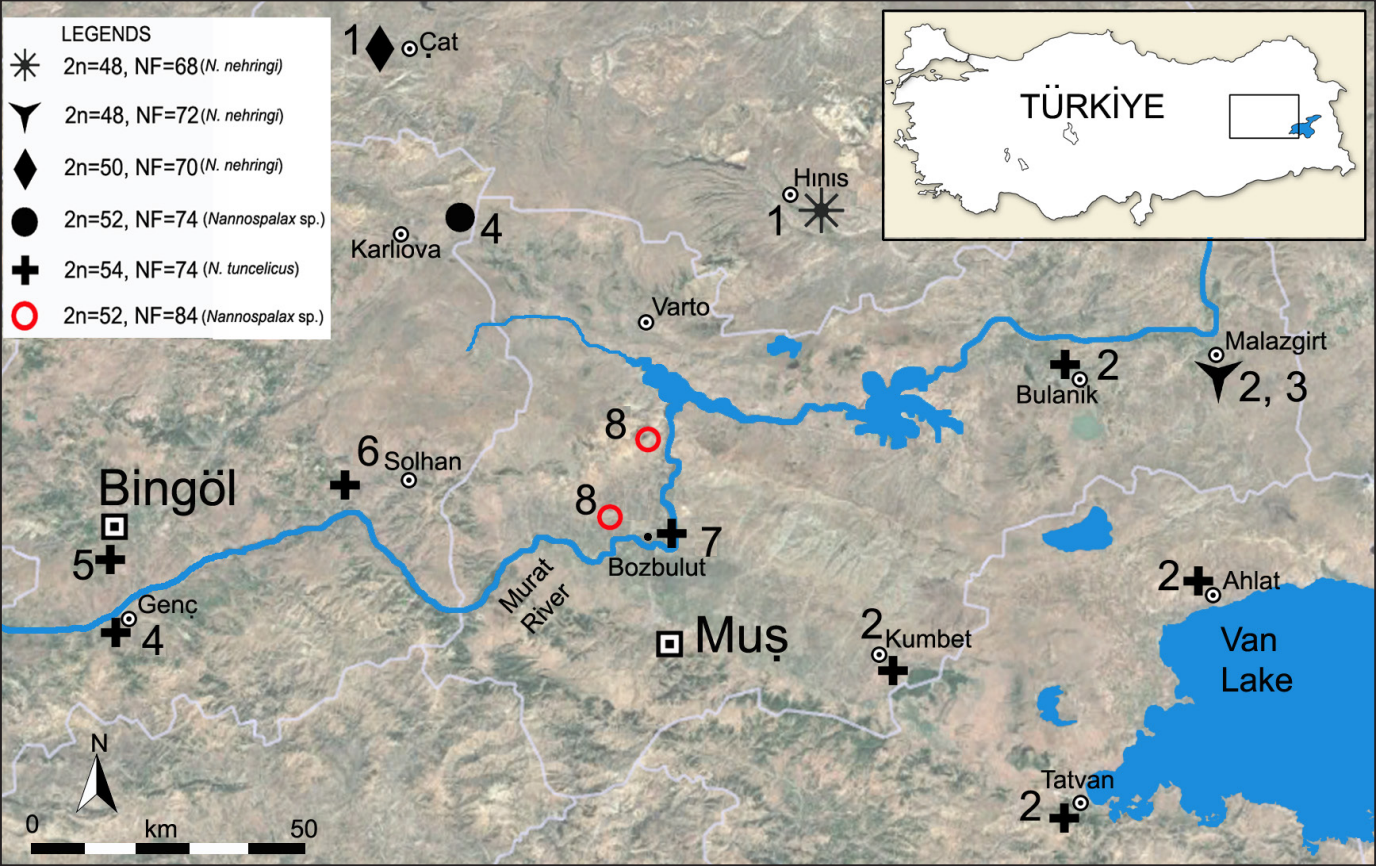
Sampling localities and karyotypes of blind mole rat specimens from the Muş Province of Türkiye. The numbers indicate the references (1. Coşkun et al. 2012; 2. Coşkun et al. 2009; 3. Arslan and Zima 2017; 4. Coşkun 2010; 5. Nevo et al. 1994; 6. Coşkun 2004; 7. Kankılıç et al. 2024; 8. This study), and symbols refer to the chromosomal forms listed in Table 1.

The study region and its surroundings exhibit significant chromosomal diversity among blind mole rats, as presented in the information above. Although more than 20 chromosomal forms of the genus *Nannospalax* have been reported in Türkiye, only those chromosomal forms recorded from Muş Province and the regions geographically closest to the current study area are included in Table 1 and Fig. 2.

**Table 1.** The chromosomal forms of *Nannospalax* in Muş Province of Türkiye and surrounding areas (2n- diploid chromosome number, NF- fundamental numbers of chromosomal arms, NFa- number of autosomal arms, m- metacentric, sm- submetacentric, st- subtelocentric, a- acrocentric) (Also see Fig. 2).

	**2n**	**NF**	**NFa**	**m, sm, st**	**a**	**X**	**Y**	**Locality**	**References**
*N. nehringi*	48	68	64	9	14	sm	a	Hınıs (Erzurum)	Coşkun et al. 2012
		72	68	11	12	sm	a	Malazgirt (Muş)	Coşkun et al. 2009; Arslan and Zima 2017
	50	70	66	9	15	sm	a	Çat (Erzurum)	Coşkun et al. 2012
*N. tuncelicus*	54	74	70	9	17	sm	a	Bingöl 10 km S	Nevo et al. 1994
								Solhan 17 km W (Bingöl)	Coşkun 2004
								Kümbet, Bulanık (Muş), Ahlat, Tatvan (Bitlis)	Coşkun et al. 2009
								Genç (Bingöl)	Coşkun 2010
								Bozbulut (Muş)	Kankılıç et al. 2024
*Nannospalax* sp.	52	74	70	10	15	m	a	Karlıova 3 km SE (Bingöl)	Coşkun 2010
		84	80	15	10	sm	a	Dilimli and Nadaslık (Muş)	**This study**

Blind mole rats are known for their extensive chromosomal variation, with many chromosomal forms proposed as cryptic species (Savić et al. 2017; Bugarski-Stanojević et al. 2022, 2024; Németh et al. 2024). However, the taxonomic status of most chromosomal forms remains unresolved and requires additional studies.

The aim of the present study is to provide new karyological and distributional data of a cryptic lineage of *Nannospalax* sp. (2n = 52) from the Eastern Anatolia region of Türkiye, which is considered to belong to the *N. nehringi* species complex.

## Material and methods

In this study, a total of five blind mole rats (*Nannospalax* sp.) from two localities of Muş Province in Türkiye were studied: three specimens (1 female, 2 male) from Dilimli village (Muş Province) (38°59.96'N, 41°31.09'E) and two specimens (2 female) from Nadaslık (Muş Province) (38°52.89'N, 41°25.91'E) (Figure 2). The animals were captured according to the method described by Zuri and Terkel (1996). The mound near the middle of the blind mole rat’s tunnel system was excavated, and the tunnel underneath was opened and properly leveled to capture the blind mole rat. When the mole rat attempted to close this opening, it was captured alive by cutting off its return path with a hoe.

Conventionally stained chromosome preparations from bone marrow were made by the standard air-drying technique (Ford and Hamerton 1956). The technique of Howell and Black (1980) was followed for detection of nucleolus organizer regions (NORs). At least 20–25 well-spread Giemsa-stained mitotic and Ag-NOR-banded plates were examined in each specimen. The diploid chromosome number (2n), the fundamental numbers of chromosomal arms (NF) and the number of autosomal arms (NFa), as well as the sex chromosomes (XX, XY) were determined. The skull and skins of specimens are deposited at the Batman University, Science and Art Faculty, Biology Department (BTU-ZL-138, BTU-ZL-139, BTU-ZL-147, BTU-ZL-148, BTU-ZL-149.

## Results

**Figure 3.**
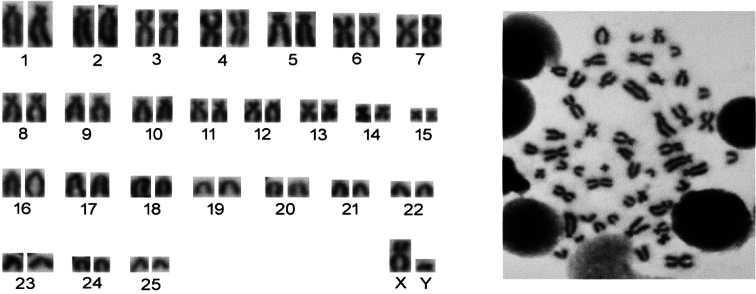
Mitotic metaphase spread and conventional stained karyotype of a male specimen of the blind mole rat from Dilimli village (Muş Province).

The karyotype of the five specimens examined in this investigation contained 2n = 52 chromosomes. The autosomal set have 15 pairs (1–15) of biarmed (m, sm and/or st), and 10 pairs of acrocentrics (16–25) of gradually diminishing size. The fundamental chromosome number was 84, and the number of autosomal arms was 80. The X chromosome was medium-sized and submetacentric while the Y chromosome was small and acrocentic (Fig. 3).

**Figure 4.**
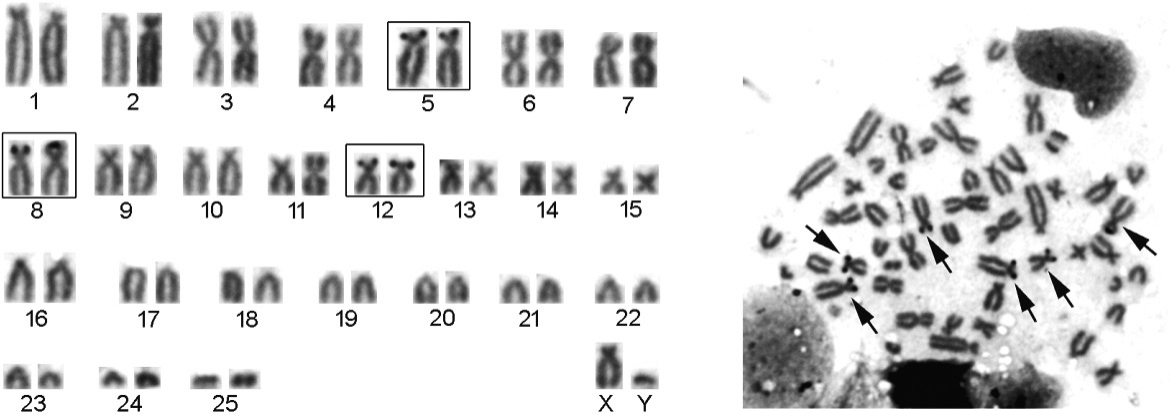
Silver nitrate-stained metaphase spread and NORs in the male karyotype of the blind mole rat from Dilimli village (Muş Province) (Arrows indicate the terminal localization of NORs).

Active NORs were found on three bi-armed autosomal pairs (no. 5, 8 and 12) in all examined specimens. The NORs were located on telomeric regions of short arms of these chromosomes (Fig. 4).

## Discussion

Chromosomal investigations play an important role in explaining the speciation and interrelationship of Spalacidae species (Gülkaç and Küçükdumlu 1999). To date, the existence of mole rat populations with varying chromosomal numbers (2n = 44, NF = 73; 2n = 48, NF = 68 and NF = 72; 2n = 49, NF = 76; 2n = 50, NF = 70 and NF = 72; 2n = 52, NF = 74; 2n = 54, NF = 74; 2n = 58, NF = 66 and NF = 68) has been reported in the Eastern Anatolia region of Türkiye (Sözen et al. 2000; Coşkun 2003, 2004; Kankılıç et al. 2007a, 2007b, Coşkun et al. 2009; Coşkun 2010; Coşkun et al. 2010, 2012; Arslan and Zima 2013, 2017; Kankılıç et al. 2017; Coşkun et al. 2018; Kankılıç et al. 2022, 2024).

Coşkun (1996) described the blind mole rat population from Tunceli as a new subspecies, *Spalax nehringi tuncelicus*, based on morphological characteristics. Later, Coşkun (2004) revised its classification using karyological data and renamed this taxon *Nannospalax tuncelicus*, also reporting that this population (2n = 54, NF = 74) is distributed in Tunceli, Elazığ, and Bingöl. Coşkun et al. (2009) classified the Malazgirt (Muş) population (2n = 48 and NF = 72) as *N. nehringi*, whereas Arslan and Zima (2017) classified it as *N. xanthodon*.

Coşkun (2010) revealed a chromosomal form of 2n = 52 (NF = 74, NFa = 70) from 3 km northeast of Karlıova (Bingöl Province, Türkiye). Our findings are consistent with those of Coşkun (2010) regarding the diploid chromosome number, while there are significant differences in terms of the number of chromosomal arms (NF and NFa). Coşkun et al. (2009) stated the karyotype of blind mole rat specimens from Bulanık and Kümbet (Muş) as 2n = 54, NF = 74. Similarly, Kankılıç et al. (2024) reported the karyotype with 2n = 54 and NF = 74 (*N. tuncelicus*) from Bozbulut village (Muş Province, Türkiye) (see Figure 2), which is located rather close to the locality studied in the present study.

Arslan and Zima (2017) observed that NORs are located in the telomeric regions of the short arms of two pairs of bi-armed chromosomes in Malazgirt (Muş Province) blind mole rats (2n = 48, NF = 72). Arslan et al. (2023) reported that the NORs were observed in the telomeric regions of the short arms of two autosomes in Bitlis blind mole rat populations (2n = 54, NF = 74).

Blind mole rats are known for their extensive chromosomal variation, with many chromosomal forms proposed as cryptic species (Savić et al. 2017; Bugarski-Stanojević et al. 2022, 2024; Németh et al. 2024). Chromosomal rearrangements (such as Robertsonian fusions, centromeric shifts, and pericentric inversions) are considered the main mechanism of speciation in rodents (Castiglia 2013). Such rearrangements are common in the chromosomal evolution of the genus *Nannospalax* (Savić et al. 2017). The differences in NF values between the 2n = 52 (NF = 84) karyotype identified in this study and the previously reported 2n = 52 (NF = 74) karyotype may indicate the occurrence of pericentric inversions or other chromosomal rearrangements affecting arm numbers. Due to the lack of studies on the NOR characteristics of the 2n = 52 (NF = 74) population previously reported by Coşkun (2010), a comparison could not be conducted.

The geographical distribution of chromosomal forms of blind mole rats in the studied area is presented in Figure 2. The map shows a virtually continuous distribution of the 54-chromosome karyotype in the southern part of the area studied, and consistent changes in the karyotype in the direction of decreasing 2n values from 54 to 52, 50, with the lowest 2n value of 48 in the northern and eastern locations. Along with changes in the number of chromosomes, those karyotypes also differ in the variability of chromosome morphology, as indicated by the fundamental and autosomal number of arms, NF and NFa. In addition, the differences in the arm numbers are revealed between the karyotypes with the same diploid number 2n = 52, the one which we described, in comparison with the other previously studied karyotype (Table 1). The NF and NFa values determined in this study of blind mole rat populations from Dilimli and Nadaslık (Muş) are among the highest recorded for blind mole rats distributed in Türkiye.

In conclusion, this study presents new karyological and distributional data on a cryptic *Nannospalax* sp. (2n = 52, NF = 84) considered belonging to the *N. nehringi* species complex in the Eastern Anatolia region of Türkiye, thereby contributing to the existing literature on the chromosomal diversity and distribution of blind mole rats in the region. Mountainous landscapes and fragmented habitats in this region may have facilitated such chromosomal divergence by limiting gene flow between populations, indicating that chromosomal divergence may have occurred in geographically isolated populations.

Confirming the species status of the populations with varied karyotypes requires comprehensive integrative taxonomic approaches combining cytogenetic, molecular phylogenetic, morphometric, and ecological data.

## Ethical approval

Ethical approval was obtained from Batman University Ethics Committee (approval date: 17.12.2025 and permission of ethics document number: E-28962623-204.01.07-249353). All applicable national guidelines for the care and use of animals were followed.
